# Iterated crowdsourcing dilemma game

**DOI:** 10.1038/srep04100

**Published:** 2014-02-14

**Authors:** Koji Oishi, Manuel Cebrian, Andres Abeliuk, Naoki Masuda

**Affiliations:** 1 Department of Applied Physics, Graduate School of Engineering, The University of Tokyo, 7-3-1 Hongo, Bunkyo, Tokyo 113-8656, Japan; 2 CREST, JST, 4-1-8 Honcho, Kawaguchi, Saitama 332-0012, Japan; 3 National Information and Communications Technology Australia, University of Melbourne, Victoria 3010, Australia; 4 Department of Mathematical Informatics, The University of Tokyo, 7-3-1 Hongo, Bunkyo, Tokyo 113-8656, Japan

## Abstract

The Internet has enabled the emergence of collective problem solving, also known as crowdsourcing, as a viable option for solving complex tasks. However, the openness of crowdsourcing presents a challenge because solutions obtained by it can be sabotaged, stolen, and manipulated at a low cost for the attacker. We extend a previously proposed crowdsourcing dilemma game to an iterated game to address this question. We enumerate pure evolutionarily stable strategies within the class of so-called reactive strategies, i.e., those depending on the last action of the opponent. Among the 4096 possible reactive strategies, we find 16 strategies each of which is stable in some parameter regions. Repeated encounters of the players can improve social welfare when the damage inflicted by an attack and the cost of attack are both small. Under the current framework, repeated interactions do not really ameliorate the crowdsourcing dilemma in a majority of the parameter space.

Crowdsourcing has opened a plethora of possibilities for individuals around the world to connect, coordinate, and solve complex problems that are currently beyond computational capabilities^1^,^2^,^3^,^4^,^5^,^6^,^7^,^8^,^9^,^10^,^11^,^12^,^13^,^14^,^15^. At the same time, a number of problems have arisen by the use of this novel technology. In particular, the openness of crowdsourcing presents individuals with an opportunity to exhibit antisocial behavior such as plagiarizing, sabotaging, and manipulating the solution being collectively obtained (see Refs. 16 and 17 for brief reviews).

Although techniques for securing crowdsourcing operations have been expanding steadily, so has the number of applications of crowdsourcing^18^. As a result, a silver bullet to secure crowdsourcing for all possible attacks may be difficult to find. Services such as Amazon's Mechanical Turk will likely diminish the problem of intentional attacks by using a reputation system, discouraging participants to sabotage^12^. Other approaches such as error correction have also been shown to be effective in crowdsourcing settings. However, they are limited in their applicability to specific contexts^19^. In this study, we consider the possibility that repeated encounters between the same peers may alleviate sabotage.

Motivated in part by the DARPA Network Challenge^9^,^10^, a crowdsourcing dilemma game in which two competing firms interact in a two-stage game was recently proposed^16^. In the first stage, each of the two firms selects whether or not to achieve a given task via crowdsourcing. If the firm decides not to crowdsource, it tries to solve the problem in-house. In the second stage, the firms have the option of attacking the opponent if the opponent has selected to solve the task via crowdsourcing. The equilibrium strategies of the model depend on complex tradeoffs between the productivity value, the benefit of attack, and the cost of attack. In summary, there are three parameter regions. First, crowdsourcing by both agents is the unique equilibrium when the damage inflicted by an attack is low. Second, the in-house solution (i.e., not crowdsourcing) selected by both agents is the unique equilibrium when the damage inflicted by an attack is high and the cost of attack is low. Third, the crowdsourcing by both agents and the in-house solution of both agents are two equilibria when the damage inflicted by an attack is high and the cost of attack is high.

In the crowdsourcing dilemma game^16^, attacking the opponent's task that has been crowdsourced lessens the welfare of both parties, which is a social dilemma. A decreased level of attacks is considered to be socially desirable. In the theory of cooperation in social dilemma situations, there have been proposed various mechanisms to evade socially undesirable equilibria. One such mechanism is iterated interaction, also called direct reciprocity. Mutual cooperation emerges in the iterated prisoner's dilemma under appropriate conditions if players adopt conditional strategies such as variants of Tit-for-Tat (i.e., do what the opponent did in the last round)^20^,^21^,^22^,^23^,^24^,^25^.

In the present study, we explore the possibility that the crowdsourcing dilemma is alleviated by similar repeated encounters between players in crowdsourcing competitions. We formulate a variant of the crowdsourcing dilemma game as an iterated game. First, we identify evolutionarily stable strategies (ESSs) of the non-iterated game as a baseline. Then, we turn to the iterated game to examine a full range of conditional strategies to identify all ESSs. Our main question is whether or not there exist conditional ESSs that outperform unconditional ESSs for given parameter values of the model. For a computational reason, we restrict ourselves to the strategies that use the information about the action of the opponent in the previous encounter.

## Results

### Model

To examine ESSs, we consider an infinitely large well-mixed population of players in which two randomly selected players are engaged in an iterated crowdsourcing dilemma game. Each player is engaged in the game sufficiently many times in one generation.

**Figure 1:**
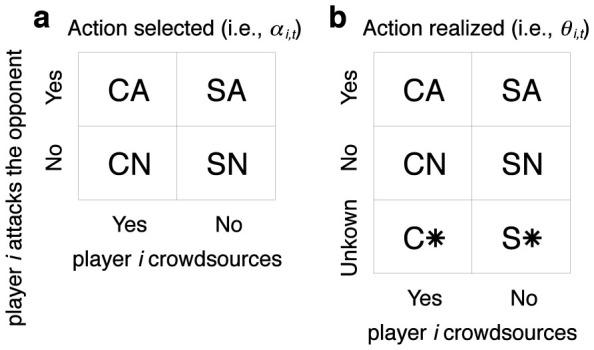
Actions selected and realized in a single round. (a) Four types of actions selected by a player in a single round. (b) Six types of actions realized by a player in a single round.

Consider the iterated game between players 1 and 2. In every round of the game, each player submits an action, which generally depends on the action of the opponent in the previous round. We denote the action selected by player *i* ∈ {1, 2} in round *t* by *α_i_*_,*t*_, which is either CA, CN, SA, or SN (Figure 1(a)). With *α_i_*_,*t*_ = CA (*α_i_*_,*t*_ = SA), player *i* selects to crowdsource (not to crowdsource) and attack the opponent if the opponent crowdsources in round *t*. With *α_i_*_,*t*_ = CN (*α_i_*_,*t*_ = SN), player *i* selects to crowdsource (not to crowdsource) and not to attack the opponent if the opponent crowdsources in round *t*. It should be noted that CA and CN are behaviorally the same unless the opponent crowdsources. In this case, the opponent that has not crowdsourced does not know whether the focal player has selected CA or CN. By the same token, SA and SN are the same unless the opponent crowdsources.

There are six types of action that a player *i* realizes in a single round (Figure 1(b)). We denote the realized action of player *i* in round *t* by *θ_i_*_,*t*_, which is either CA, CN, C*, SA, SN, or S*. *θ_i_*_,*t*_ = CA (*θ_i_*_,*t*_ = SA) means that player *i* has crowdsourced (has not crowdsourced) and attacked the opponent. *θ_i_*_,*t*_ = CN (*θ_i_*_,*t*_ = SN) means that player *i* has crowdsourced (has not crowdsourced) and has not attacked the opponent. *θ_i_*_,*t*_ = C* (*θ_i_*_,*t*_ = S*) means that player *i* has crowdsourced (has not crowdsourced) and that whether player *i* has intended to attack the opponent or not is unknown to the opponent. If *θ_i_*_,*t*_ is either CA, CN, SA, or SN, *i*'s opponent has crowdsourced in round *t*. If *θ_i_*_,*t*_ is either C* or S*, *i*'s opponent has not crowdsourced. The relationship between the actions selected by the two players and the realized actions perceived by the two players is shown in Table 1.

We consider players adopting the so-called reactive strategies^22^,^23^,^25^,^26^. A player adopting a reactive strategy selects an action based on the opponent's realized action in the previous round. Therefore, a reactive strategy of player 1 is a mapping from *θ*_2,*t*_ to *α*_1,*t*+1_. There are 4^6^ = 4096 reactive strategies.

We assume that players commit an action implementation error with a small probability 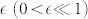. For simplicity, the decision of crowdsourcing and that of attacking are assumed to err independently with the same probability 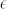. For example, a player intending CA actually carries out CA with probability 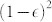, CN with probability 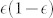, SA with probability 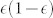, and SN with probability 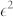.

The payoff in a round is determined in the same way as in the original crowdsourcing dilemma game^16^. A player's productivity value is equal to zero as normalization when the player does not crowdsource. It obeys the uniform distribution on (0, 1) when the player crowdsources. A player needs to pay cost *q* ∈ (0, 1) to attack the opponent to reduce the opponent's productivity by *d* ∈ (0, 1). The player that finally obtains the higher productivity than the opponent wins the unitary payoff in the current round. The other player gains nothing. If the productivity values of the two players are the same, each player wins with probability 1/2. It should be noted that players decide the actions without referring to the productivity values of the player itself and the opponent.

We do not consider time discounting of the payoff across rounds and do assume that the number of rounds is very large. Therefore, we are concerned with the stationary state of the actions adopted by the two players and the payoff per round.

Even if we confine ourselves to a single-round game, the present model is slightly different from the previous model^16^ in the following aspects. First, in the previous model^16^, it was assumed that the productivity values of both players thanks to crowdsourcing were unknown to each player when the players determined whether to crowdsource or not in the first stage. The productivity values were then revealed just before the second stage occurred. In other words, if the opponent has crowdsourced, the focal player knows the opponent's productivity (and the focal player's own productivity if the focal player has crowdsourced) before they determine whether to attack the opponent or not. Therefore, each player is assumed to obey the best response rule in the second stage. In contrast, in the present model, we assumed that the players select the actions for the first stage (i.e., crowdsource or not to crowdsource) and the second stage (i.e., attack or not to attack) in the beginning of the round without knowing the productivity of the players in the middle of the round. We changed the model in this way because, otherwise, there are a continuum of pure strategies because of the productivity is continuously valued. By confining ourselves to a model with a finite set of discrete pure strategies, we aim to carry out an exhaustive and rigorous analysis of the model to understand the iterated as well as non-iterated crowdsourcing dilemma game.

### Non-iterated game

**Figure 2:**
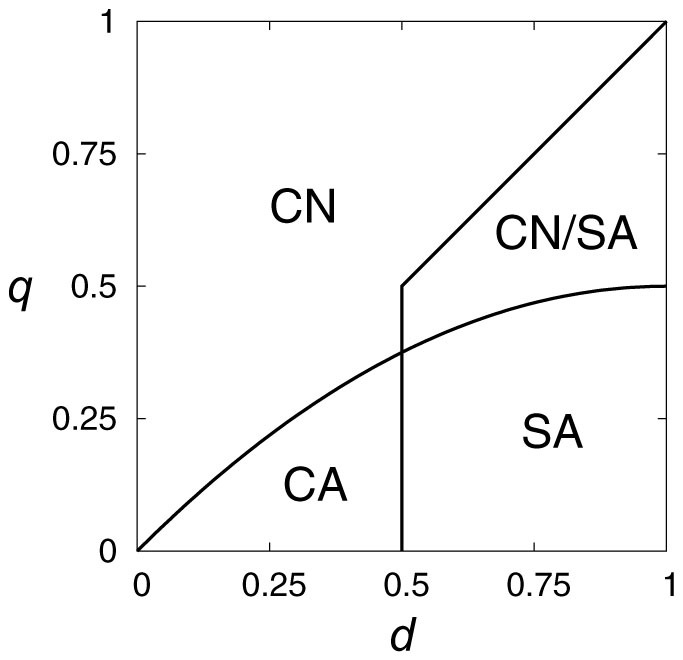
Phase diagram for the single-shot game. In the region labeled CN/SA, both CN and SA are ESSs.

We started by analyzing the non-iterated crowdsourcing dilemma game. Because strategies conditioned on the realized action in the previous round are irrelevant, there are four pure strategies, i.e., CA, CN, SA, and SN. The ESSs in the full (*d*, *q*) parameter space are shown in Figure 2. The figure indicates that crowdsourcing is stable when the damage inflicted by an attack (i.e., *d*) is small or the cost of attack (i.e., *q*) is large. Attacking is stable when *d* is large or *q* is small.

The results shown in Figure 2 are qualitatively the same as those for the previously analyzed single-shot crowdsourcing dilemma game^16^ in the meaning that crowdsourcing is stable when *d* is small or *q* is large. In contrast, a large *q* value does not prevent the players from attacking the opponent in the previous model^16^, whereas not to attack is an ESS for large *q* (irrespectively of *d*) in the present model.

### Evolutionary stability and efficiency for the iterated game

**Figure 3:**
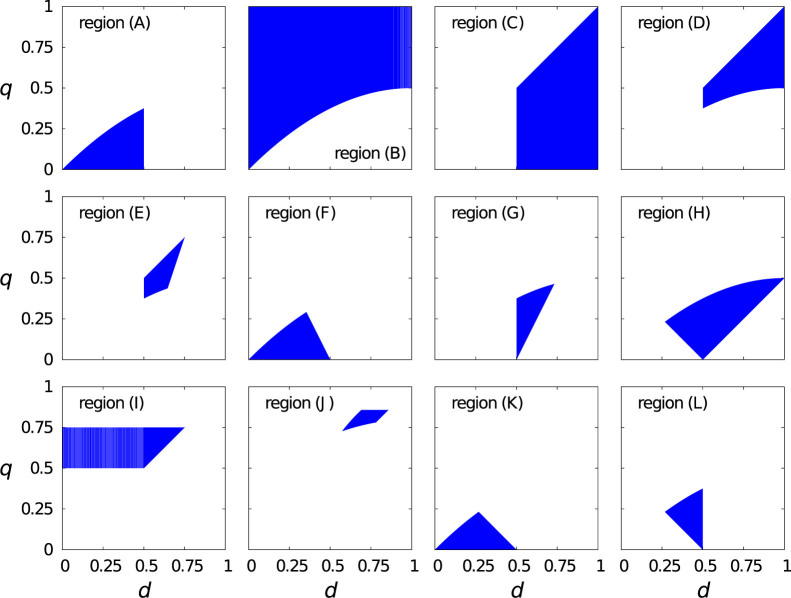
Parameter regions (A) to (L) in the (*d*, *q*) space. In addition to the trivial condition 0 < *d*, *q* < 1, the 12 regions are defined as follows. Region (A): *d* < 1/2 and *q* < (1/2)*d*(2 − *d*). Region (B): *q* > (1/2)*d*(2 − *d*). Region (C): *d* > 1/2 and *q* < *d*. Region (D): *d* > 1/2 and (1/2)*d*(2 − *d*) < *q* < *d*. Region (E): *d* > 1/2 and max{(1/2)*d*(2 − *d*), (3/2)(2*d* − 1)} < *q* < *d*. Region (F): *q* < min{(1/2)*d*(2 − *d*), 1 − 2*d*}. Region (G): *d* > 1/2 and 2*d* − 1 < *q* < (1/2)*d*(2 − *d*). Region (H): max{1/2 − *d*, *d* − 1/2} < *q* < (1/2)*d*(2 − *d*). Region (I): max{*d*, 1/2} < *q* < 3/4. Region (J): max{(2/5)(1 + 2*d* − *d*^2^), *d*} < *q* < min{(1/2)(−1 + 6*d* − 3*d*^2^), 6/7}. Region (K): *q* < min{(1/2)*d*(2 − *d*), 1/2 − *d*}. Region (L): *d* < 1/2 and 1/2 − *d* < *q* < (1/2)*d*(2 − *d*).

We exhaustively searched ESSs among the 4096 reactive strategies. We found 16 strategies that were ESSs in some regions of the (*d*, *q*) parameter space. The 16 ESSs are listed in Table 2. In the table, *α_n_*(SN), for example, indicates the action selected when the opponent realized SN in the previous round. Each strategy is an ESS in the parameter region specified by the label (one of (A) through (J)) shown in the table. The parameter regions are depicted in Figure 3 (see the caption for the precise definition).

Ten out of the 16 ESSs are efficient for some *d* and *q* values. The parameter regions in which these ESSs are efficient are shown in Table 2. We checked the condition for the efficiency by referring to the average payoffs of ESSs in the homogeneous population (Table 3).

Strategies 1, 2, and 3 are unconditional strategies, whereas the other strategies are conditional strategies. We call the three unconditional strategies uncond-CA, uncond-CN, and uncond-SA, respectively.

In parameter regions (B) and (C), uncond-CN and uncond-SA are efficient ESSs, respectively. This result is the same as that for the single-shot game (Figure 2). In the intersection of regions (B) and (C), which is region (D), strategies 4, 5, 6, 7, 8, and 9 are also efficient ESSs. In subregions of (B) and (C), strategies 10, 11, 13, 15, and 16 are inefficient ESSs. Strategies 10, 11, and 15, but not 13 yield the same payoff as the efficient ESSs in the limit 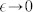. Strategy 13 is the only ESS that yields a smaller payoff than that of the coexisting unconditional ESS (i.e., uncond-SA) in the limit 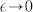.

In region (A), neither uncond-CN nor uncond-SA is an ESS, and uncond-CA is an inefficient ESS. Instead, strategy 12 or 14, both of which are conditional strategies, is the efficient ESS in the region. It should be noted that, in the single-shot game, (uncond-)CA is the unique ESS in this parameter region (Figure 2). Because regions (A), (B), and (C) exhaust the entire parameter space 0 < *d* < 1, 0 < *q* < 1, conditional ESSs yield larger payoffs than unconditional ESSs only in region (A). In other words, making the crowdsourcing dilemma game an iterated game improves the efficiency of the ESS exclusively in this parameter region.

Region (A) is composed of subregions (K) and (L).

In region (K), strategy 14 is not an ESS, and strategy 12 is the efficient ESS. The payoff of strategy 12 in the homogeneous population is larger than that of uncond-CA by 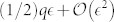. The difference vanishes in the limit 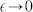. This is because a pair of players adopting strategy 12 almost always implements CA for infinitesimally small 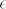.

In spite of this similarity between strategy 12 and uncond-CA, strategy 12 is efficient because, when a pair of players adopts strategy 12, their realized actions persist in SA for some time once both players start implementing SA. To understand this phenomenon, consider the situation in which both players adopting strategy 12 implement CA. This situation almost always occurs in the limit 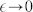. The two players simultaneously switch to SA if both players commit an error to select either SA or SN in the same round. This event occurs with probability 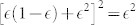. They return to selecting CA if either player commits an error to select CA or CN. This event occurs with probability 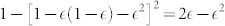. Therefore, the fraction of the number of rounds in which the two players implement SA is approximately equal to 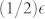 for small 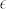. During the period in which the two players implement SA, the cost of attack is evaded. In contrast, a player adopting uncond-CA cannot avoid the cost of attack, irrespective of whether the opponent adopts uncond-CA or strategy 12. This is because the repetition of SA does not persist and both players almost always implement CA. Therefore, strategy 12 is stable against invasion by uncond-CA and yields a slightly larger payoff than uncond-CA in the homogeneous population.

In region (L), strategy 14 is the efficient ESS. The payoff of strategy 14 in the homogeneous population is larger than that of uncond-CA by 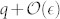, which does not vanish for infinitesimally small 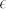.

A pair of players adopting uncond-CA almost always implements CA and obtains 1/2 − *q* per round for infinitesimally small 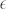. This is because both players pay the cost of attack (i.e., *q*) and win the game with probability 1/2. In contrast, a pair of players adopting strategy 14 almost always implements SA for infinitesimally small 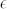, as shown in Table 3. The two players obtain 1/2 per round; each player wins with probability 1/2 without paying the cost of attack. This is the reason why strategy 14 yield a larger payoff than uncond-CA in the limit 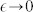. A player adopting strategy 14 alternates between CA and SA in the absence of error if the opponent adopts uncond-CA. In this situation, the average payoff of the opponent is equal to (1/2) {(1 − *d*) + (1/2 − *q*)}. Therefore, strategy 14 is stable against invasion by uncond-CA when *q* > 1/2 − *d*. This condition defines a boundary of region (L). It should be noted that, when *d* < 1/2, i.e., when being attacked is not so costly, players gain a larger payoff by selecting CA rather than SA irrespective of the action of the opponent. Therefore, in contrast to strategy 14, uncond-SA is not stable in region (L).

### Size of attractive basins of different ESSs in parameter region (A)

**Figure 4:**
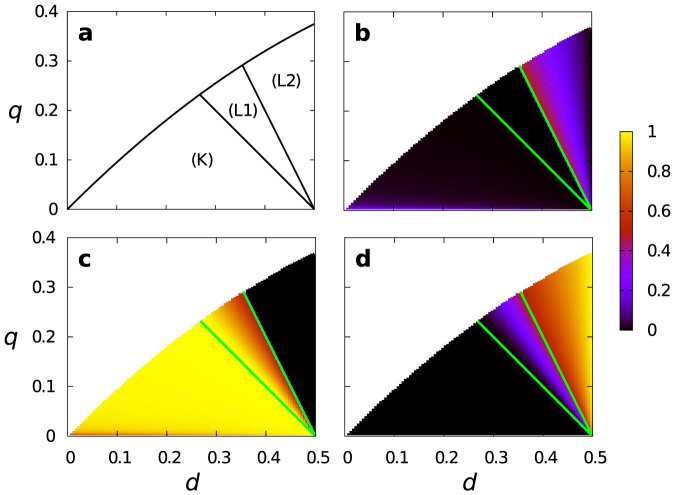
Details of region (A). (a) Three subregions of region (A) in the (*d*, *q*) space. (b), (c), (d) Relative sizes of the attractive basins. (b) uncond-CA, (c) strategy 12, and (d) strategy 14. We set 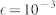. The two solid lines in (b), (c), and (d) represent the boundaries between the subregions. The size of attractive basin of strategies 12 and 14 is equal to zero in region (L2) and region (K), respectively, because they are not ESSs in the corresponding region.

In the previous section, we revealed that conditional strategies were the only efficient ESSs in region (A). For these conditional strategies to establish a foothold in an evolutionary context, they should also have a sufficiently large attractive basin under evolutionary dynamics. Therefore, we compare the relative size of the attractive basins of the ESSs in region (A). We examine replicator dynamics composed only of ESSs because it is not feasible to treat the dynamics composed of all 4096 strategies. Region (K) allows two ESSs, i.e., uncond-CA and strategy 12. Region (L) is divided into subregion (L1) that allows three ESSs, i.e., uncond-CA, strategy 12, and strategy 14, and subregion (L2) that allows two ESSs, i.e., uncond-CA and strategy 14. The boundary between (L1) and (L2) is given by *q* = 1 − 2*d*. These regions within region (A) are depicted in Figure 4(a). We separately calculated the size of attractive basins for regions (K), (L1), and (L2).

The size of the attractive basin for each ESS is shown in Figure 4(b)--(d) with 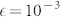. The attractive basins of strategies 12 and 14 are larger than that of uncond-CA for a large parameter region in region (A). In particular, strategies 12 and 14 have the largest attractive basin in most of region (K) and the entire region (L2), respectively. Therefore, conditional strategies 12 and 14 are not only efficient but also reached from various initial conditions under replicator dynamics.

## Discussion

We explored the possibility of improving the quality of the solution in a crowdsourcing game by analyzing an iterated game. We found that the crowdsourcing dilemma was alleviated when the damage inflicted by an attack (i.e., *d*) and the cost of attack (i.e., *q*) were small (i.e., region (A)). Our main conclusions are as follows: (i) In parameter region (A), an unconditional strategy (uncond-CA) and either conditional strategy (strategy 12 or 14) are coexisting ESSs. (ii) Furthermore, the conditional strategies 12 and 14 are more efficient than uncond-CA in region (A). (iii) Outside region (A), repeated encounters do not alter the efficient ESSs relative to the case of the non-iterated game.

Strategy uncond-CA is analogous to unconditional defection in the prisoner's dilemma game. Strategies 12 and 14 are analogous to retaliative strategies in the prisoner's dilemma game. However, we emphasize that, the loose analogue between the crowdsourcing dilemma game and the prisoner's dilemma game is only justified in region (A). Because strategy 12 is only marginally superior to uncond-CA in region (A), we discuss the combat between strategy 14 and uncond-CA; the homogeneous population of strategy 14 yields the payoff that is larger by ≈ *q* than that realized by the homogeneous population of uncond-CA. If strategy 14 and uncond-CA play the iterated game, each strategist almost always selects CA or SA. In our game, CA and SA are analogous to defection and cooperation in the prisoner's dilemma, respectively. If both selects SA, both players obtain 1/2 in a single round if 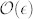 terms are neglected. If the focal player selects SA and the opponent selects CA, the focal player gains *d* − *q*. If the focal player selects CA and the opponent selects SA, the focal player gains 1 − *d*. If both players select CA, both players obtain 1/2 − *q*. Because 1 − *d* > 1/2 > 1/2 − *q* > *d* − *q* and 2 × (1/2) > (1 − *d*) + (*d* − *q*), the single-shot crowdsourcing dilemma game played by strategy 14 and uncond-CA is essentially the same as the prisoner's dilemma.

The uncond-CA strategy is equivalent to the unconditional defection in the prisoner's dilemma. To describe the behavior of the player adopting strategy 14, we refer to such a player simply as strategy 14 here. If strategy 14 realizes SA and the opponent realizes SA, corresponding to mutual cooperation, strategy 14 selects SA (i.e., cooperation) in the next round. If strategy 14 realizes SA and the opponent realizes CA, strategy 14 is exploited by the opponent and switches to CA (i.e., defection) in the next round. If strategy 14 realizes CA and the opponent realizes SA, strategy 14 exploits the opponent and continues to select CA. If strategy 14 realizes CA and the opponent realizes CA, corresponding to mutual defection, strategy 14 switches to SA. Therefore, strategy 14 is equivalent to the win-stay lose-shift strategy in the iterated prisoner's dilemma^23^,^24^. Our results pertaining to the improved efficiency of strategy 14 relative to uncond-CA are consistent with the results obtained for the win-stay lose-shift strategy in the iterated prisoner's dilemma^23^,^24^.

Intuitively, both crowdsourcing and not attacking are expected to be analogous to cooperation, and not crowdsourcing and attacking are expected to be analogous to defection. However, the present model as well as the previous one^16^ do not allow the association between crowdsourcing (not crowdsourcing) and cooperation (defection) because of the definition of the payoff. In both models, the winning player gains a payoff equal to unity. Given that attacking does not occur, crowdsourcing increases the probability of winning owing to the enhanced productivity. However, whether the solution is made in-house or by crowdsourcing does not affect the payoff in any other way. For example, if the realized actions of both players are SN (i.e., not crowdsourcing and not attacking), each player gains an expected payoff equal to 1/2. If the realized actions of the two players are CN (i.e., crowdsourcing and not attacking), the payoff remains the same. However, in real situations, crowdsourcing is considered to improve the quality of the solution unless an attack occurs^3^,^5^,^11^. To examine non-iterated and iterated crowdsourcing dilemma games with this added component warrants future work. In this study, we confined ourselves to a simpler scenario, thus avoiding to introduce yet a new parameter.

## Methods

### Calculation of average payoffs

Throughout the present paper, we concentrate on the set of pure reactive strategies. We calculate the average payoff of player 1 that adopts reactive strategy *n* when the opponent player 2 adopts reactive strategy *m*, denoted by *π_nm_*.

In each round, there are nine possible pairs of actions realized by the two players. In other words, (*θ*_1,*t*_, *θ*_2,*t*_) is either (CA,CA), (CA,CN), (CN,CA), (CN,CN), (C*,SA), (C*,SN), (SA,C*), (SN,C*), or (S*,S*). Given (*θ*_1,*t*_, *θ*_2,*t*_), the actions that the two players intend to carry out in the next round are determined by *n* and *m*. Then, we calculate the probability with which each pair of actions (*α*_1,*t*+1_, *α*_2,*t*+1_) is actually selected. This probability depends on 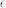. Then, the two players play the game such that a pair of realized actions (*θ*_1,*t*+1_, *θ*_2,*t*+1_) is uniquely determined from the pair of selected actions (*α*_1,*t*+1_, *α*_2,*t*+1_), as shown in Table 1. By combining the stochastic mapping from (*θ*_1,*t*_, *θ*_2,*t*_) to (*α*_1,*t*+1_, *α*_2,*t*+1_) and the deterministic mapping from (*α*_1,*t*+1_, *α*_2,*t*+1_) to (*θ*_1,*t*+1_, *θ*_2,*t*+1_), we obtain the transition probability from (*θ*_1,*t*_, *θ*_2,*t*_) to (*θ*_1,*t*+1_, *θ*_2,*t*+1_). Any (*θ*_1,*t*+1_, *θ*_2,*t*+1_) is reached from any (*θ*_1,*t*_, *θ*_2,*t*_) with a positive probability because of the error 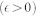. Therefore, the Markov chain on (*θ*_1,*t*_, *θ*_2,*t*_) is ergodic and possesses a unique stationary distribution.

The average payoff of player 1 is given by 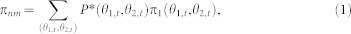
where P*(*θ*_1,*t*_, *θ*_2,*t*_) is the probability that (*θ*_1,*t*_, *θ*_2,*t*_) is realized in the stationary state, and *π*_1_(*θ*_1,*t*_, *θ*_2,*t*_) is the expected payoff of player 1 under (*θ*_1,*t*_, *θ*_2,*t*_). Table 4 shows the values of *π*_1_(*θ*_1,*t*_, *θ*_2,*t*_). It should be noted that if both players crowdsource, player 1 attacks player 2, and player 2 does not attack player 1 (i.e., (*θ*_1,*t*_, *θ*_2,*t*_) = (CA, CN)), then player 1 wins if *p*_1_ > *p*_2_ − *d*, where *p*_1_ and *p*_2_ are productivity values of players 1 and 2, respectively. This event occurs with probability 1 − (1/2)(1 − *d*)^2^.

### Evolutionary stability and efficiency

Strategy *n* is an ESS if *π_nn_* > *π_mn_* is satisfied or both *π_nn_* = *π_mn_* and *π_nm_* > *π_mm_* are satisfied for all *m* ≠ *n*. We enumerate all ESSs as follows using essentially the same exhaustive search method as that used for studying indirect reciprocity^27^.

Consider strategy *n*. For all strategies *m* ≠ *n*, we check the following conditions. If *π_nn_* and *π_mn_* are not the same function in terms of *d*, *q*, and 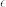, we expand the difference with respect to 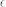 as follows: 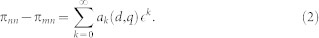
We denote the nonzero coefficient of the lowest order on the right-hand side of Eq. (2) by 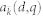. For infinitesimally small 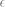, strategy *n* is stable against invasion by strategy *m* if 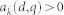. If *π_nn_* and *π_mn_* are the same function, then we compare *π_nm_* and *π_mm_* with the same procedure. If *π_nm_* and *π_mm_* are the same function, strategy *n* is not an ESS because it is neutrally stable against invasion by strategy *m*. If *n* is not invaded by any *m*, even neutrally, *n* is an ESS.

We say that ESS *n* is efficient if *π_nn_* ≥ *π_mm_* for all other ESSs *m*(≠ *n*). To check the efficiency of ESSs, we expand *π_nn_* − *π_mm_* with respect to 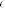 and look at the sign of the non-zero coefficient of the lowest order.

### Calculation of the size of the attractive basin under replicator dynamics

We denote the frequency of players adopting strategy *n* by *x_n_* ∈ [0, 1]. The replicator equation is given by 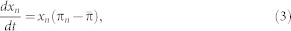
where 
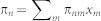
 and 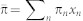.

Consider the case in which just two pure-strategy ESSs, denoted by *n* = 1 and 2, exist. Then, under the replicator dynamics composed of two strategies 1 and 2, the relative size of the attractive basin of strategy 1 is given by (*π*_11_ − *π*_21_)/(*π*_11_ + *π*_22_ − *π*_12_ − *π*_21_).

Consider the case in which three pure-strategy ESSs, denoted by *n* = 1, 2, and 3, exist. Then, we assume a population composed of the three ESSs and determine the basin size of the ESSs by direct numerical integration of the replicator equation because analytical expressions are difficult to obtain. We run the dynamics from initial conditions (*x*_1_, *x*_2_, *x*_3_) = (*ℓ*_1_Δ, *ℓ*_2_Δ, *ℓ*_3_Δ), where Δ = 1/200, and (*ℓ*_1_, *ℓ*_2_, *ℓ*_3_) is a set of integers that satisfy 0 < *ℓ*_1_, *ℓ*_2_, *ℓ*_3_ < 200 and *ℓ*_1_ + *ℓ*_2_ + *ℓ*_3_ = 200. We count the number of initial conditions such that all players finally adopt strategy 1 and divide it by the total number of initial conditions. The calculated fraction defines the relative size of the attractive basin of strategy 1. Parallel definitions are applied to strategies 2 and 3.

## Author Contributions

M.C. and N.M. designed the research; K.O. contributed the computational results; K.O., M.C., A.A. and N.M. discussed the results; K.O., M.C., A.A. and N.M. wrote the paper.

## Figures and Tables

**Table 1:** Relationship between the actions selected by the two players and the realized actions

*α*_1,*t*_	*α*_2,*t*_	*θ*_1,*t*_	*θ*_2,*t*_
CA	CA	CA	CA
CA	CN	CA	CN
CA	SA	C*	SA
CA	SN	C*	SN
CN	CA	CN	CA
CN	CN	CN	CN
CN	SA	C*	SA
CN	SN	C*	SN
SA	CA	SA	C*
SA	CN	SA	C*
SA	SA	S*	S*
SA	SN	S*	S*
SN	CA	SN	C*
SN	CN	SN	C*
SN	SA	S*	S*
SN	SN	S*	S*

**Table 2:** ESSs. *α_n_*(*θ*) is the action that a player with strategy *n* selects when the opponent's realized action was *θ* in the previous round. The regions for ESS and efficiency in the table indicate the parameter regions in which the strategy is an ESS and an efficient ESS, respectively. See Figure 3 for the definition of the region labels. If an ESS is efficient nowhere, the entry for the region for efficiency remains blank

Strategy (*n*)	*α_n_*(CA)	*α_n_*(CN)	*α_n_*(C*)	*α_n_*(SA)	*α_n_*(SN)	*α_n_*(S*)	Region for ESS	Region for efficiency
1 (uncond-CA)	CA	CA	CA	CA	CA	CA	(A)	
2 (uncond-CN)	CN	CN	CN	CN	CN	CN	(B)	(B)
3 (uncond-SA)	SA	SA	SA	SA	SA	SA	(C)	(C)
4	CN	CN	CN	CN	CN	SA	(D)	(D)
5	CN	CN	SA	SA	SA	CN	(D)	(D)
6	CN	CN	SA	SA	SA	SA	(D)	(D)
7	SA	SA	CN	CN	CN	CN	(D)	(D)
8	SA	SA	CN	CN	CN	SA	(D)	(D)
9	SA	SA	SA	SA	SA	CN	(D)	(D)
10	CN	SA	SA	SA	SA	SA	(D)	
11	CN	SA	CN	CN	CN	SA	(E)	
12	CA	CA	CA	CA	CA	SA	(F)	(K)
13	SA	SA	CA	CA	CA	CA	(G)	
14	SA	SA	CA	CA	CA	SA	(H)	(L)
15	SN	CA	CA	CA	CA	SN	(I)	
16	SN	CN	CA	CA	CA	SN	(J)	

**Table 3:** Stationary distribution of the selected actions of two players adopting an ESS, in the limit 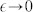. *P*(*α*) is the probability that both players select action *α*. The probability that the two players select different actions tends to zero as 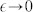. The payoff when both players adopt the same strategy is also shown

Strategy (*n*)	*P*(CA)	*P*(CN)	*P*(SA)	*P*(SN)	Payoff (*π_nn_*)
1 (uncond-CA)	1	0	0	0	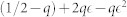
2 (uncond-CN)	0	1	0	0	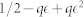
3 (uncond-SA)	0	0	1	0	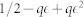
4	0	1	0	0	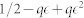
5	0	1	0	0	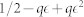
6	0	0	1	0	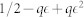
7	0	1/2	1/2	0	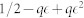
8	0	0	1	0	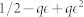
9	0	1/2	1/2	0	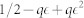
10	0	0	1	0	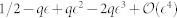
11	0	0	1	0	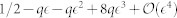
12	1	0	0	0	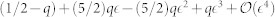
13	1/2	0	1/2	0	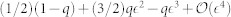
14	0	0	1	0	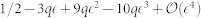
15	0	0	0	1	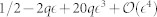
16	0	0	0	1	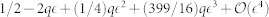

**Table 4:** The expected payoff *π*_1_(*θ*_1,*t*_, *θ*_2,*t*_) of player 1 when the realized actions of player 1 and 2 are *θ*_1,*t*_ and *θ*_2,*t*_, respectively

*θ*_1,*t*_	*θ*_2,*t*_	*π*_1_(*θ*_1,*t*_, *θ*_2,*t*_)
CA	CA	1/2 − *q*
CA	CN	1 − (1/2)(1 − *d*)^2^ − *q*
CN	CA	(1/2)(1 − *d*)^2^
CN	CN	1/2
C*	SA	1 − *d*
C*	SN	1
SA	C*	*d* − *q*
SN	C*	0
S*	S*	1/2

## References

[b1] Howe J. The rise of crowdsourcing. Wired Mag. 14, 1–4 (2006).

[b2] von Ahn L. Games with a purpose. Computer 39, 92–94 (2006).

[b3] von Ahn L., Maurer B., McMillen C., Abraham D. & Blum M. re-CAPTCHA: Human-based character recognition via web security measures. Science 321, 1465–1468 (2008). 18703711 10.1126/science.1160379

[b4] Huberman B. A., Romero D. M. & Wu F. Crowdsourcing, attention and productivity. J. Inform. Sci. 35, 758–765 (2009).

[b5] Cooper S. *et al.* Predicting protein structures with a multiplayer online game. Nature 466, 756–760 (2010). 20686574 10.1038/nature09304 PMC2956414

[b6] Hand E. *et al.* Citizen science: People power. Nature 466, 685–687 (2010). 20686547 10.1038/466685a

[b7] Horowitz D. & Kamvar S. D. The anatomy of a large-scale social search engine., in Proc. 19th ACM International Conference on World Wide Web, 431–440 (2010).

[b8] Hellerstein J. M. & Tennenhouse D. L. Searching for Jim Gray: A technical overview. Comm. ACM 54, 77–87 (2011).

[b9] Pickard G. *et al.* Time-critical social mobilization. Science 334, 509–512 (2011). 22034432 10.1126/science.1205869

[b10] Tang J. C. *et al.* Reflecting on the DARPA red balloon challenge. Comm. ACM 54, 78–85 (2011).

[b11] Barrington L., Turnbull D. & Lanckriet G. Game-powered machine learning. Proc. Natl. Acad. Sci. U.S.A. 109, 6411–6416 (2012). 22460786 10.1073/pnas.1014748109 PMC3340027

[b12] Mason W. & Suri S. Conducting behavioral research on Amazon's Mechanical Turk. Behav. Res. Methods 44, 1–23 (2012). 21717266 10.3758/s13428-011-0124-6

[b13] Zhang H., Horvitz E., Chen Y. & Parkes D. C. Task routing for prediction tasks., in Proc. 11th International Conference on Autonomous Agents and Multiagent Systems 2, 889–896 (2012).

[b14] Alstott J., Madnick S. & Velu C. Measuring and predicting speed of social mobilization. arXiv:1303.3805 (2013).

[b15] Rahwan I. *et al.* Global manhunt pushes the limits of social mobilization. Computer 46, 68–75 (2013).

[b16] Naroditskiy V., Jennings N. R., Hentenryck P. V. & Cebrian M. Crowdsourcing dilemma. arXiv:1304.3548 (2013).

[b17] Watts D., Cebrian M. & Elliot M. Dynamics of social media., in Public Response to Alerts and Warnings Using Social Media: Report of a Workshop on Current Knowledge and Research Gaps, 22–33, eds. National Research Council (The National Academies Press, Washington, D.C., 2013).

[b18] Kittur A. *et al.* The future of crowd work., in Proc. 2013 Conference on Computer Supported Cooperative Work, 1301–1318 (2013).

[b19] Ipeirotis P. G., Provost F., Sheng V. S. & Wang J. Repeated labeling using multiple noisy labelers. Data Min. Knowl. Disc. 28, 402–441 (2014).

[b20] Trivers R. L. The evolution of reciprocal altruism. Quart. Rev. Biol. 46, 35–57 (1971).

[b21] Axelrod R. The Evolution of Cooperation (Basic Books, NY, 1984).

[b22] Nowak M. A. & Sigmund K. Tit for tat in heterogeneous populations. Nature 355, 250–253 (1992).

[b23] Kraines D. & Kraines V. Learning to cooperate with Pavlov–an adaptive strategy for the iterated Prisoner's Dilemma. Theor. Decis. 35, 107–150 (1993).

[b24] Nowak M. A. & Sigmund K. A strategy of win-stay, lose-shift that outperforms tit-for-tat in the Prisoner's Dilemma game. Nature 364, 56–58 (1993). 8316296 10.1038/364056a0

[b25] Nowak M. A. Evolutionary Dynamics (Harvard Univ. Press, Cambridge, 2006).

[b26] Nowak M. A. & Sigmund K. The evolution of stochastic strategies in the Prisoner's Dilemma. Acta Appl. Math. 20, 247–265 (1990).

[b27] Ohtsuki H. & Iwasa Y. How should we define goodness–reputation dynamics in indirect reciprocity. J. Theor. Biol. 231, 107–120 (2004). 15363933 10.1016/j.jtbi.2004.06.005

